# MgO Modified by X_2_, HX, or Alkyl Halide (X = Cl, Br, or I) Catalytic Systems and Their Activity in Chemoselective Transfer Hydrogenation of Acrolein into Allyl Alcohol

**DOI:** 10.3390/molecules29133180

**Published:** 2024-07-03

**Authors:** Marek Gliński, Urszula Ulkowska, Zbigniew Kaszkur, Dariusz Łomot, Piotr Winiarek

**Affiliations:** 1 Faculty of Chemistry, Warsaw University of Technology, Noakowskiego 3, 00-664 Warsaw, Poland; urszula.ulkowska@pw.edu.pl (U.U.); piotr.winiarek@pw.edu.pl (P.W.); 2 Institute of Physical Chemistry, Polish Academy of Sciences, Kasprzaka 44/52, 01-224 Warsaw, Poland; zkaszkur@ichf.edu.pl (Z.K.); dlomot@ichf.edu.pl (D.Ł.)

**Keywords:** magnesium oxide, halogens, transfer hydrogenation, acrolein, allyl alcohol

## Abstract

A new type of catalyst containing magnesium oxide modified with various modifiers ranging from bromine and iodine, to interhalogen compounds, hydrohalogenic acids, and alkyl halides have been prepared using chemical vapor deposition (CVD) and wet impregnation methods. The obtained systems were characterized using a number of methods: determination of the concentration of X^−^ ions, surface area determination, powder X-ray diffraction (PXRD), surface acid–base strength measurements, TPD of probe molecules (acetonitrile, pivalonitrile, triethylamine, and *n*-butylamine), TPD-MS of reaction products of methyl iodide with MgO, and Fourier transform infrared spectroscopy (FTIR). The catalysts’ activity and chemoselectivity during transfer hydrogenation from ethanol to acrolein to allyl alcohol was measured. A significant increase in the activity of modified MgO (up to 80% conversion) in the transfer hydrogenation of acrolein was found, while maintaining high chemoselectivity (>90%) to allyl alcohol. As a general conclusion, it was shown that the modification of MgO results in the suppression of strong basic sites of the oxide, with a simultaneous appearance of Brønsted acidic sites on its surface. Independently, extensive research on the reaction progress of thirty alkyl halides with MgO was also performed in order to determine its ability to neutralize chlorinated wastes.

## 1. Introduction

Magnesium oxide is known as the one of the most frequently used metal oxides in heterogeneous catalysis [1]. In contrast to other commonly used oxides, such as Al_2_O_3_, Al_2_O_3_-SiO_2_, SiO_2_, TiO_2_, and zeolites, the surface of MgO possesses strong basic properties [2]. The process of alkylation of phenol with methanol to 2,6-xylenol, which was developed by General Electric in the 1970s, is the leading commercial application of magnesium oxide as a catalyst [3]. Furthermore, magnesium oxide is used as an active catalyst in a double C=C bond migration (SHOP process) and in aldol condensation reactions of carbonyl compounds, nitroaldol condensation, and Michael addition [2]. Very recently, this oxide has been used as a catalyst for the transesterification of triglicerides with methanol [4,5]. It is also widely applied in the catalytic transfer hydrogenation of various carbonyl compounds with alcohols [6,7]. The following carbonyl compounds have been studied: aralkyl ketones [8], cycloalkanones [9], aliphatic ketones with diverse steric hindrances [10], and a derivative of cyclohexanone for which diastereoselectivity of the reduction of a carbonyl group could be determined [11]. The strong basic properties of the surface of MgO (H_-_ < 33) [2] can be either beneficial or a disadvantage depending on the chemical needs of the reactants. For specific reactions, the basicity of MgO is not strong enough and must be strengthened through the deposition of alkali metals onto its surface to reach the level of basicity expressed by a value of H_-_ < 35 [12]. For other reactions, the basicity of MgO itself is too high and must be lowered via various treatments such as impregnation with inorganic acids [13], *n*-butyl iodide [14], or chloroderivatives of methane [15,16,17]. This can also be achieved through the appropriate choice of a precursor of MgO and a method of oxide preparation that suppresses the basicity of the final product [18]. In 1974, Kibblewhite and Tench reported that gaseous halogens (Cl_2_, Br_2_ and I_2_) react at room temperature with magnesium oxide. The extent of the oxide substitution on the resulting surface strongly depends on the type of the halogen [19]. It has been shown that MgO treated with halogens contains the appropriate X^−^ anions and lacks XO^−^ and XO_3_^−^ anions, and that those treated with Cl_2_ or Br_2_ release oxygen when heated. No evolution of oxygen is observed for the MgO-I_2_ system, although I^−^ ions are present on the surface of MgO. The differences between the values of the enthalpies of formation of solid MgO and MgX_2_ (X = Cl, Br, I) indicate that chlorine, and to a lesser extent bromine, are capable of replacing oxide ions in the MgO lattice. According to the authors, iodine can only react with the surface oxide ions of the lowest coordination, e.g., 3-fold coordination, which are the most reactive. Flockhardt et al. published the results of studies on the reduction of iodine by the surfaces of Al_2_O_3_, Al_2_O_3_-SiO_2_, and SiO_2_ in a benzene solution [20]. The authors stated that the hydroxyl groups on the surfaces of the metal oxides can act as one-electron donor sites, and that they are responsible for the occurrence of the reaction. The authors have proposed the mechanism of the reduction in accordance with the following equation (Equation (1)).

(1) $$I_{2}+2{OH}^{-}\to {IO}^{-}+I^{-}+H_{2}O$$

However, they could not explain the absence of IO_3_^−^ ions, derived from the transformations of IO^−^ ions, which should be formed as per Equation (2).

(2) $$3{IO}^{-}\to {IO}_{3}^{-}+2I^{-}$$

In our former work, we reinvestigated the system of MgO-I_2_ studied by Flockhardt et al. [20] and found an explanation for the above-described discrepancy [21]. Furthermore, we demonstrated that MgO treated with a solution of iodine in 2-pentanol, various alkyl iodides, or chloroderivatives of methane shows a significant increase in the selectivity in liquid-phase hydrogen transfer to cyclopentanone [9,22]. Similar systems, in which MgO was modified with halogens, have been shown to exhibit halogenating properties [23], noticeable biocidal activity [24], and to catalyze the oxidative dehydrogenation of butane to butadiene [25], as well as the dehydrohalogenation of different chlorobutane isomers or 1-bromobutane [26]. 

Based on the results of our previous studies, we concluded that MgO–halogen systems still have significant potential, offering attractive opportunities in emerging new areas, primarily in terms of applications in heterogeneous catalysis. Therefore, one of the aims of this study was to conduct extensive research using a range of MgO surface modifiers, such as halogens, interhalogen compounds, hydrohalic acids, alkyl halides, etc., to obtain new catalytic systems for the transfer hydrogenation of acrolein. This molecule is the simplest α,β-unsaturated aldehyde, which is one of the most difficult compounds to chemoselectively reduce to the unsaturated alcohol [27]. In our previous study on the activity of pure MgO in the catalytic transfer hydrogenation (CTH) of acrolein with alcohols, we have shown that modification of its surface could be beneficial in terms of the chemoselectivity of this reaction at moderate temperatures [28]. Since the MgO-I_2_ system has already shown very promising catalytic properties in other reactions [15,17,22,25], in which the molecule did not allow for the assessment of chemoselectivity, this specific parameter was the main focus of the presented research. This study also entails an investigation of the reactions of the bromine or iodine vapor phase or dissolved in different alcohols with magnesium oxide, which were carried out across a broad range of temperatures, to probe their potential for chlorinated waste treatment. 

## 2. Results

**Scheme 1:**
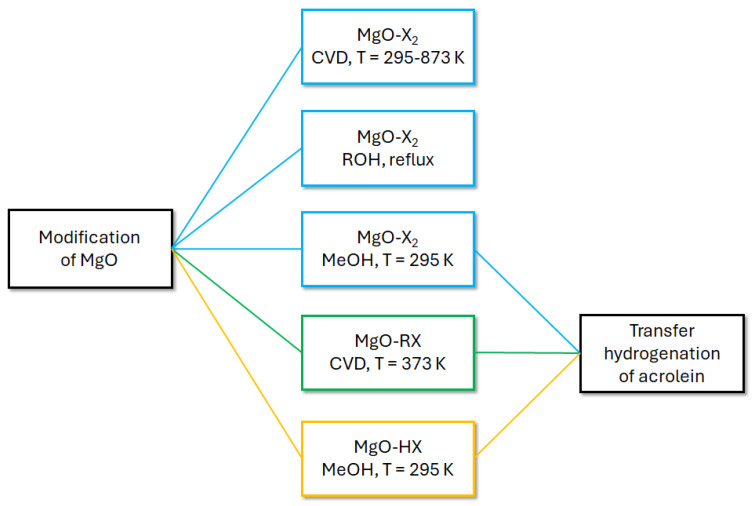
Generalized reaction diagram.

Magnesium oxide was treated with a number of modifiers: halogens (Br_2_ or I_2_) in the vapor phase or in solution in various alcohols, alkyl halides in the vapor phase, or hydrohalic acids in methanol at room temperature. Most of the new synthesized catalytic systems were subjected to activity tests in the vapor phase transfer hydrogenation reaction of acrolein with ethanol. For an easier overview of the research process in this work, the types of modifiers, the conditions of their reaction with MgO, and the types of catalysts tested in the above-mentioned reaction are summarized in the diagram below (Scheme 1). 

### 2.1. Reaction of MgO with Bromine or Iodine in Vapor Phase or in Solution with Various Alcohols

In our former work, we have shown that magnesium oxide reacts with iodine in the vapor phase or in solution in a nonpolar solvent (cyclohexane) under anhydrous conditions. The formation of iodide ions occurs to a very small extent, i.e., below 25 μmol per 1 g of oxide [21]. We have also found that no IO_n_^−^ ions were formed. The consumption of iodine can be described by Equation (3). The source of electrons transferred to iodine is not established. It is postulated in literature that they are derived from surface hydroxyls and/or the lowest coordinated oxygen anions acting as one-electron donor sites [19,29].

(3) $$I_{2}+2\bar{e}\to 2I^{-}$$

In the current work, we extended our research to the reactions of iodine with MgO in various alcohols both at room temperature and at their boiling point, with the expectation that, under these conditions, the reactions would proceed with higher yields. We also included bromine in our studies, which is a more reactive halogen than iodine. The first step in this area was to investigate the reactivity of bromine in a reaction with magnesium oxide over a range of temperatures of 295–873 K and compare the obtained results with those previously obtained for iodine under the same conditions [21]. The results are summarized in Table 1. It was found that part of the bromine introduced into the reaction was reduced to bromide ions on the MgO surface. In the range of 295–873 K, their concentration depended very little on the reaction temperature and amounted to 115 μmol g^−1^ MgO at the highest temperature. This value is over five times higher than the analogous value of the iodide ion concentration (22 μmol g^−1^ MgO) obtained for the reaction of iodine with MgO at the same temperature [21]. Moreover, due to the much higher volatility of bromine compared to iodine, the presence of bromine in the samples starting at 473 K was not observed.

In order to increase the concentration of iodide ions on the MgO surface, studies were carried out on the reaction of iodine with the oxide in alcohols at their boiling points. Several aliphatic alcohols were selected, differing in order, including methanol, ethanol, and a sequence of three secondary alcohols: 2-propanol, 2-pentanol, 3-pentanol—as well as a tertiary alcohol, *t*–butanol (Table 2 and Table 3). No quantitative conversion of iodine was observed for any alcohol, despite 6 h of heating at reflux. The highest concentration of iodide ions was obtained in the case of 2-pentanol; their concentration reached 12.1 mmol g^−1^ for MgO. The lowest concentration was 6.5 mmol g^−1^ obtained in *t*-butanol. No iodate ions were found in any of the post-reaction mixtures. The analysis of the iodide ion concentrations obtained in these experiments clearly indicated that the reaction of MgO with iodine was not limited only to the oxide surface (i.e., not limited to reaction with Mg^2+^_surf_). If only the surface magnesium ions reacted with iodine, the ratio of iodide ions to magnesium cations would be 2.0. However, the values of this ratio noted in the conducted research ranged from 3.5 to 6.5, which indicates additional I^−^–Mg^2+^_bulk_ interactions.

Studies of the composition of the post-reaction liquids using GC-MS provided new, important information about the course of the reaction and the roles of the alcohols in it. It was found that, depending on the order of alcohol used in the synthesis, various organic products were formed. For ethanol, 1,1-diethoxyethane was the main product, and both acetaldehyde and ethyl acetate were noted as minor products. When using secondary alcohols as solvents/reagents, the formation of the corresponding ketones was detected, in addition to secondary alkyl iodides and the corresponding ethers. The presence of products with a structure other than those described above was observed in the case of a tertiary alcohol, *t*-butanol. The dominant product was methylpropene, with a minor presence of 2,4,4-trimethyl-1-pentene and *t*-butyl *i*-butyl ether.

This work postulates that regardless of the order of the alcohol, the first four steps of the reaction of the iodine with the alcohol are the same. In line with the view expressed in the literature [30,31], the reaction begins with the attack of iodine on alcohol with the formation of alkyl hypoiodite and hydrogen iodide according to Equation (4). Next, the resulting HI is removed through the reaction with magnesium oxide (Equation (5)). The alkyl hypoiodite is then transformed into an aldehyde or ketone, depending on the order of the alcohol used, combined with the elimination of the hydrogen iodide molecule (Equations (6) and (7)). The aldehyde (but not the ketone) formed reacts with the alcohol to generate the hemiacetal (Equation (8)). The latter compound reacts with iodine in the next step (Equation (9)). The final product of the transformation is an ester, formed in accordance with Equation (10).

(4) $${RCH}_{2}OH+I_{2}\to {RCH}_{2}OI+HI$$

(5) $$MgO+HI\to Mg\left(OH\right)I$$

(6)
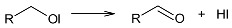

(7)
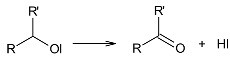

(8)
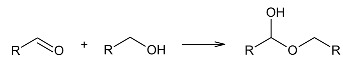

(9)
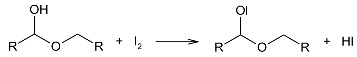

(10)
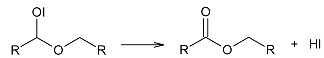



The presence of alkyl iodides obtained from the reaction of iodine with secondary alcohols was also found in the post-reaction mixture. However, the presence of methyl, ethyl, and *t*-butyl iodides was not observed. Direct iodination of alcohols occurs only in the case of secondary, tertiary, and benzyl alcohols [30]. The lack of *t*-butyl iodide can be explained by its high reactivity and the course of the hydrogen iodide elimination reaction in a basic (MgO and Mg(OH)I) reaction medium. This reaction is confirmed by the presence of alkenes in the post-reaction mixture: methylpropene and 2,4,4-trimethyl-1-pentene. The presence of ethers in the post-reaction mixture is the result of the Williamson etherification reaction, according to Equation (11):
(11)
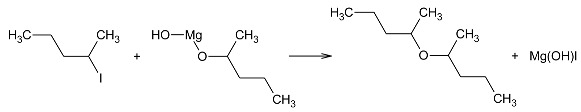


Two alcohols that exhibit peculiar behavior in the reaction with iodine are ethanol and methanol. Following the formation of the hemiacetal (Equation (8)), the main organic product of ethanol transformations in the studied reaction, namely 1,1-diethoxyethane, is formed as a product of acetaldehyde acetalization. The ease of formation of acetals by aldehydes is undoubtedly a factor favoring this reaction. A certain mechanistic difficulty in the formation of this compound is the fact that its synthesis requires the participation of an acid catalyst. It can be assumed that the HI produced in earlier stages is responsible for the course of this reaction.

In the case of methanol as a reagent, no organic compounds resulting from its transformation with iodine and magnesium oxide were found in the post-reaction mixture. The presence of the following compounds was excluded: methanal, 1,3,5-trioxane, 1,1-dimethoxymethane, methyl formate, trimethyl orthoformate, tetramethyl orthocarbonate, methyl iodide, methylene iodide, and triiodomethane or any other organic iodine-containing compounds. However, there was no doubt that methanol was a reagent in this reaction, not just a solvent, which was confirmed by the high concentration of iodide ions obtained. Based on the above premises, the mechanism of methanol transformation in the reaction was proposed. In the first two stages, similarly to other alcohols, unstable methyl hypoiodite is formed, which decomposes to formaldehyde. The HI produced in these reactions reacts with MgO to form hydroxomagnesium iodide. In the third step, the aldehyde undergoes a Canizzaro reaction to form methanol and hydroxomagnesium formate. Under the reaction conditions, it is also possible to proceed to the next, fourth stage, in which the obtained hydroxomagnesium formate is oxidized with the iodine remaining in the mixture with the release of carbon dioxide and the formation of hydroxomagnesium iodide. Indeed, the oxidation of formates (Equation (12)) with iodine is used for the analytical quantification of formates [32]. The end products of the transformation sequence are hydroxomagnesium iodide, hydroxomagnesium formate, and carbon dioxide.

(12) $$HCOONa+I_{2}\to NaI+HI+{CO}_{2}$$

Due to the selectivity of methanol transformations in the tested reaction, including the absence of organic compounds containing iodine (alkyl iodides), as well as the associated losses of iodine and organic compounds with relatively high boiling points, methanol was selected as the reagent for further research. The reaction of iodine with MgO in methanol at room temperature was studied for two initial concentrations of iodine (Table 4). The reactions were carried out without stirring to avoid attrition of the MgO used in the reaction and in the dark to avoid photolytic decomposition of methyl hypoiodite. It was found that for the lower initial iodine concentration, the reaction in methanol was completed within 10 h, whereas at an iodine concentration of 3.00 mmol g^−1^ of MgO, the reaction ended after reaching the final iodide concentration of 1.35 mmol g^−1^ of MgO after 40 h. The same studies using bromine as a reagent led to different conclusions (Table 5). Bromine reacts much faster with MgO in methanol than does iodine. For both of its concentrations, complete consumption of the halogen in the reaction was observed.

### 2.2. Vapor Phase Reaction of Various Alkyl Halides with MgO

Magnesium oxide is a strong basic oxide, and this property can be used to neutralize acidic and/or chlorinated wastes. At the beginning of the twenty-first century, novel methods for the neutralization of chlorinated waste were developed by Klabunde et al. using nanocrystalline MgO as a neutralizing agent. They used the oxide for the dehydrohalogenation of *n*-butyl chloride, bromide, and iodide [14,24,25]. We decided to investigate the reaction of MgO with different alkyl halides (chlorides, bromides, and iodides in Table 6, Table 7, and Table 8, respectively) in order to determine the effectiveness of the dehydrohalogenation reaction depending on the type of the alkyl halide. Furthermore, we expected that the obtained new MgO-RX systems would exhibit improved catalytic activity in the tested reaction, i.e., in the chemoselective transfer of hydrogen from ethanol to acrolein with the formation of allyl alcohol.

Table 6 contains information about the concentration of Cl^−^ ions, as well as the specific surface area and color of the MgO after its reaction with a series of alkyl chlorides. All of the tested compounds reacted with magnesium oxide, which was confirmed by the analysis of the concentration of chloride ions on its surface. In the case of polyhaloderivatives of methane, the concentration of chloride ions decreased in the following order: CHCl_3_ > CH_2_Cl_2_ > CH_3_Cl > CCl_4_. What is noteworthy is that their high specific surface area was comparable to the specific surface area of the pure oxide, and all post-reaction solids were colorless like the original sample. Ethyl chloride showed a very similar reactivity in reaction with MgO to that of methyl chloride. The significantly lower specific surface area of the MgO-EtCl system compared to MgO-CH_3_Cl indicates a relevant difference in the course of these reactions in the case of both alkyl chlorides. A significant influence of the order of the alkyl chloride used on the concentration of chloride ions in the obtained systems was found. In the case of isomeric chlorobutanes, the concentration of chloride ions increased with the increase in the order of the chloroderivative. The highest concentrations of Cl^−^ ions were recorded when both tertiary derivatives (*t*-BuCl and *t*-AmCl) were used as modifiers. A comparative analysis of the concentration values of the chloride ions in the MgO-*t*-BuCl and MgO-*t*-AmCl systems, which were 19.3 and 12.2 mmol g^−1^, respectively. The concentration value of the surface <Mg-O> species in pure MgO, which is approximately 2.0 mmol g^−1^ [33], clearly indicates that the oxide in its entire volume is subjected to the attack of the modifier. The maximum achievable Cl^−^ concentration resulting from the quantitative formation of MgCl_2_ is 24.8 mmol g^−1^. As a result of the attack of the modifier (*t*-BuCl) on MgO, its specific surface area decreases significantly to only 9 m^2^ g^−1^.

Table 7 summarizes the results obtained for MgO after its reaction with selected bromides. The highest reactivity among the bromoderivatives of methane as MgO modifiers was noted for CHBr_3_, similarly to CHCl_3_ in the previous series of measurements. Small changes in the specific surface area of MgO during the reaction with the above-mentioned bromoderivatives were found. Ethyl bromide, similarly to ethyl chloride, caused a significant decrease in the specific surface area of MgO. The same dependence of the reactivity of bromoderivatives on their order as in the case of chloroderivatives was observed. *t*-butyl bromide showed the highest reactivity in the reaction with MgO. In the post-reaction sample, the concentration of bromide ions was 12.42 mmol g^−1^. The specific surface area of MgO after the reaction was only 1 m^2^ g^−1^, which indicates a substantial reconstruction of the oxide. This sample also exhibited a pronounced difference of color after the reaction.

Alkyl iodides as MgO modifiers were tested under the same conditions (373 K, time 3 h) as the two previous groups of alkyl halides (Table 8). At a temperature of 373 K, during the 3 h reaction of CH_3_I with the oxide, the concentration of iodide anions was 0.25 mmol g^−1^. The lowest concentrations of iodide anions, namely 0.08 and 0.10 mmol g^−1^, were obtained in the reaction of MgO with CH_2_I_2_ and CHI_3_. The highest concentrations of iodide ions were achieved in the case of the reaction of MgO with *s*-BuI and *t*-BuI molecules. In the case of the reaction with both of these, the concentrations of the iodide ions on the MgO surface were 0.56 and 2.33 mmol g^−1^, respectively. A significant reconstruction of the MgO structure was found after the reaction with these modifiers; the specific surface area of MgO decreased to the values of 41 and 15 m^2^ g^−1^, respectively. 

For CH_3_I, additional tests were performed under slightly different conditions (temperature and reaction time). As a result of extending the reaction time from 3 to 6 h, the concentration of iodide anions increased to 0.27 mmol g^−1^ with the simultaneous appearance of free iodine in the sample, indicating partial decomposition of the modifier. With the increase in the reaction temperature from 373 to 473 K, the concentration of iodide ions on the MgO surface was almost the same as that obtained at the lower temperature (0.24 mmol g^−1^) with a higher concentration of free iodine.

Through a thermodynamic analysis of the feasibility of the dehydrohalogenation of *n*-BuX (X = Cl, Br or I), Klabunde et al. found that *n*-BuCl should be more susceptible to HX elimination than *n*-BuBr, whereas *n*-BuI should not undergo HI elimination at temperatures below 600 °C [25]. Our results clearly show that for a low reaction temperature (373 K), the thermodynamic relationships determined by the mentioned authors do not apply. Contrary to the statement given by Klabunde et al., the least reactive *n*-butyl halide is chloride, and bromide and iodide are equally reactive.

The last groups of MgO modifiers studied were dihalides of methane and propane, as well as two interhalogen compounds (Table 9). In the reaction of bromochloromethane with MgO, bromide ions with a concentration of 0.19 mmol g^−1^ were preferentially deposited onto the oxide surface. The selectivity of the bromide attack, given as the [Br^−^]/[Cl^−^] ratio, was 3.17. In the case of ClCH_2_I as a modifier, the dominant anions on the MgO surface were chlorides, with an attack selectivity expressed by the [Cl^−^]/[I^−^] ratio equal to 5.0. Under the same reaction conditions, bromoiodomethane left almost exclusively bromide ions on the MgO surface ([Br^−^]/[I^−^] = 34.0). In the 1-bromo-3-chloropropane molecule, halogen atoms are separated by three carbon atoms, which results in a slight increase in the reactivity of chlorine towards bromine. Iodine bromide as a modifier of MgO delivers only bromide ions to the MgO surface, just as iodine trichloride does in the case of chloride ions. The results of MgO modification with bromine and iodine, added for comparison, clearly indicate their much lower reactivity in reaction with the MgO surface. The interaction of alkane dihalides as well as interhalogen compounds with MgO resulted in higher concentrations of the corresponding halide anions.

### 2.3. Characterization of MgO-X_2_, MgO-HX, and MgO-RX Systems

The obtained MgO-X_2_, MgO-HX, and MgO-RX systems were characterized using a number of methods, including specific surface area determination, powder X-ray diffraction measurements (PXRD), surface acid–base strength measurements, temperature programmed desorption of probe molecules (acetonitrile, pivalonitrile, triethylamine, and *n*-butylamine), temperature-programmed desorption of reaction products of methyl iodide with MgO, and Fourier transform infrared spectroscopy (FTIR). The results of the XPS measurements of three systems, MgO-Br_2_, MgO-I_2_, and MgO-HI, were performed, and their results have already been published by us [34,35].

#### 2.3.1. Powder X-ray Diffraction Measurements

**Figure 1:**
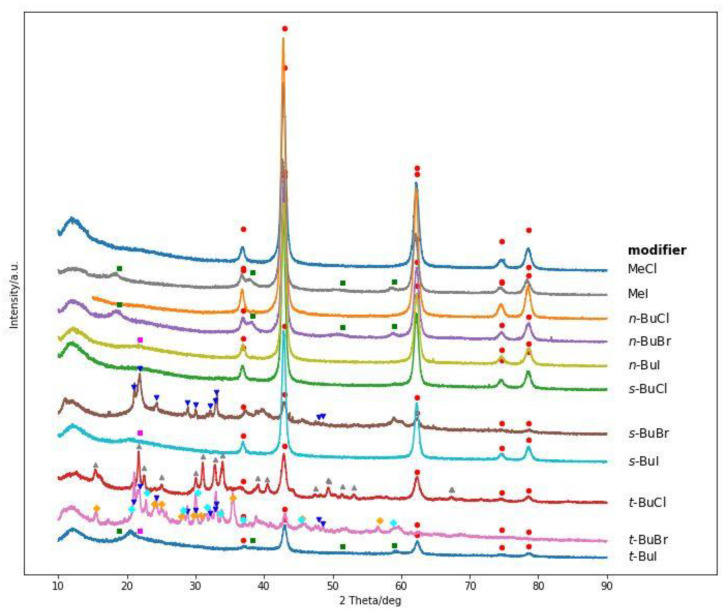
PXRD of magnesium oxide modified with various alkyl halides. ●—MgO (PDF#78-0430); ▪—Mg(OH)_2_ (PDF#86-0441); ▲—MgCl_2_∙6H_2_O (PDF#77-1268); ▼—MgBr_2_∙6H_2_O (PDF#24-0702); ◆—Mg_3_Br_2_(OH)_4_∙4H_2_O (PDF#12-0125); ◆—Mg_3_Br(OH)_6_∙4H_2_O (PDF#12-0121); ▪—MgI_2_∙6H_2_O (PDF#20-0678).

All tested magnesium oxide samples modified with selected alkyl halides contained a magnesium oxide phase. Some of them also contained Mg(OH)_2_, as well as hydrated hydroxomagnesium halides and hydrated magnesium dihalides phases (Figure 1). The appearance of the last two phases was observed only in the case of MgO-RX systems for which the concentration of halide anions was higher than 2.0 mmol g^−1^ and therefore only when the MgO modifiers were either tertiary alkyl halides (*t*-BuX and *t*-AmCl) and *s*-BuBr. It was found that in the case of modification with *t*-butyl chloride and iodide, as for MgO, the only reflections, apart from those corresponding to the MgO and Mg(OH)_2_ phases, are the reflections coming from the MgX_2_·6H_2_O phases (X = Cl or I). For *t*-butyl iodide, the diffraction pattern shows a weak reflection at a scattering angle of 21°, corresponding to the MgI_2_·6H_2_O phase. The use of *t*-butyl bromide leads to the formation of not only the hydrated magnesium dibromide phase but also phases of hydroxomagnesium bromides. Among the identified phases, reflections derived from MgBr_2_·6H_2_O exhibit the highest intensity. For the remaining MgO-RX systems, only a decrease in the intensity of reflections coming from MgO was observed in relation to the intensity of the corresponding reflections of the pure MgO phase and the appearance of low-intensity reflections coming from the formed Mg(OH)_2_ phase. Diffraction patterns containing only reflections from MgO were recorded for the MgO-HX samples (X = Cl, Br or I, [X] = 200 μmol g^−1^) and the MgO-X_2_ series (X = Br or I, [X] = 200 μmol g^−1^).

The size of crystallites in the catalyst samples analyzed using PXRD was calculated using Scherrer’s formula (for k = 1). Calculations were performed for a series of MgO-RX catalysts (Tables S1 and S2, Supplementary Materials). An increase in the size of MgO crystallites was observed after its reaction with primary and secondary BuX (X = Cl, Br or I). Modification of MgO with MeX (X = Cl, Br or I) did not affect the size of the MgO crystallites, whereas the action of *t*-BuX (X = Cl, I) resulted in a decrease in the size of the MgO crystallites. In the case of the most reactive alkyl halides, the formed MgX_2_·6H_2_O (X = Cl or Br) phases were characterized by a much larger crystallite size in the range of 22–27 nm compared to the crystallite size of pure MgO (11.9 nm).

#### 2.3.2. Strength of Acidic and Basic Site Measurements of Catalysts

The strengths of the acidic and basic sites on the surface of the MgO-X_2_, MgO-HX, and MgO-RX catalysts were measured using the Hammett indicators method. The results of these measurements are collected in Table 10, Table 11 and Table 12. On the surface of pure magnesium oxide, there are basic sites with very different strengths; the strongest ones are described with the value of the function H_-_ = 26.5, which, according to Tanabe’s definition, means that the MgO surface possesses superbasic properties [36]. As for the acidic strength of the MgO surface, it can be said that it is below the detection limit of the method, because none of the basic Hammett indicators changed their color (none were protonated) in contact with the oxide surface. In all cases, the use of modifiers in the reaction with MgO caused a decrease in the basic strength of the oxide surface with the simultaneous appearance of acidic sites on its surface. The only noted exception was CCl_4_, the use of which did not change the acid–base strength of the MgO surface. The system with the highest acidic strength was the MgO-HCl system, for which the H_0_ > −5.6 value was determined. The acidic strength of the sites described by the value of H_0_ > −3.0 was noted for the following systems: MgO-I_2_, MgO-HBr, MgO-HI, MgO-CHCl_3_, and MgO-MeI. A very significant decrease in the basic strength of the sites to the value of 7.2 ≤ H_-_ < 9.3 was noted in the case of two systems, MgO-I_2_ and MgO-MeI, and the highest decrease in the basic strength (H_-_ < 7.2) was noted for the MgO-*t*-BuCl system. In the latter case, the basic strength of the sites can be said to be below the detection limit of the Hammett indicator method, because none of the acid indicators used changed their color.

#### 2.3.3. Temperature-Programmed Desorption (TPD) of Probe Molecules

**Figure 2:**
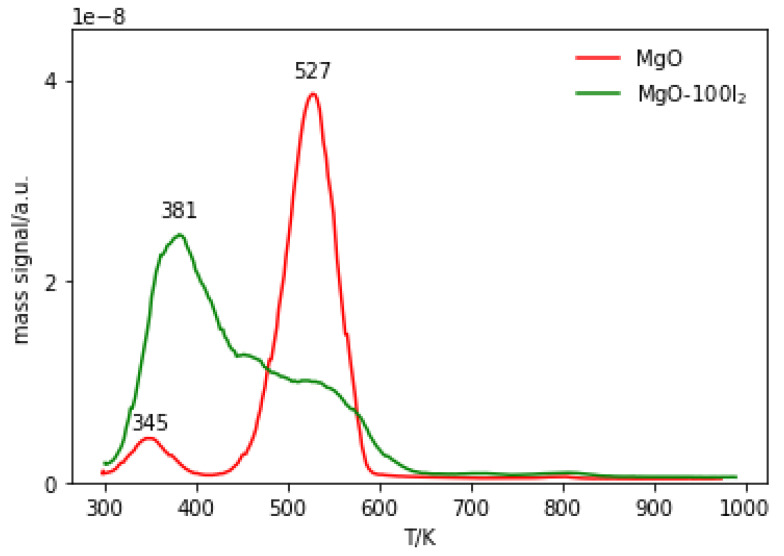
TPD of *t*-BuCN from MgO and MgO-I_2_ (MeOH).

**Figure 3:**
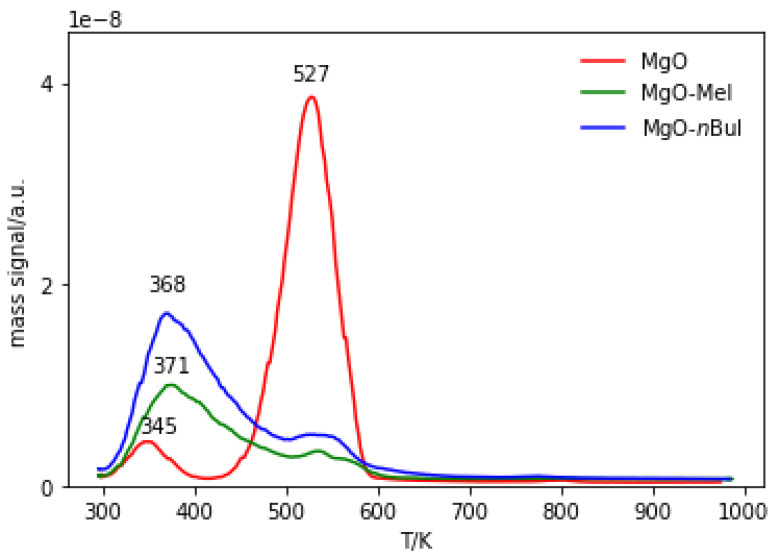
TPD of *t*-BuCN from MgO, MgO-MeI, and MgO-*n*-BuI.

**Figure 4:**
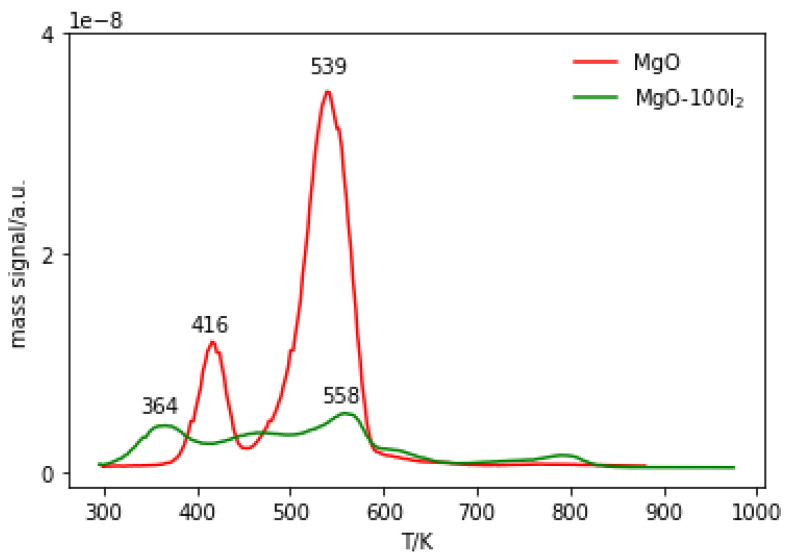
TPD of MeCN from MgO and MgO-I_2_ (MeOH).

**Figure 5:**
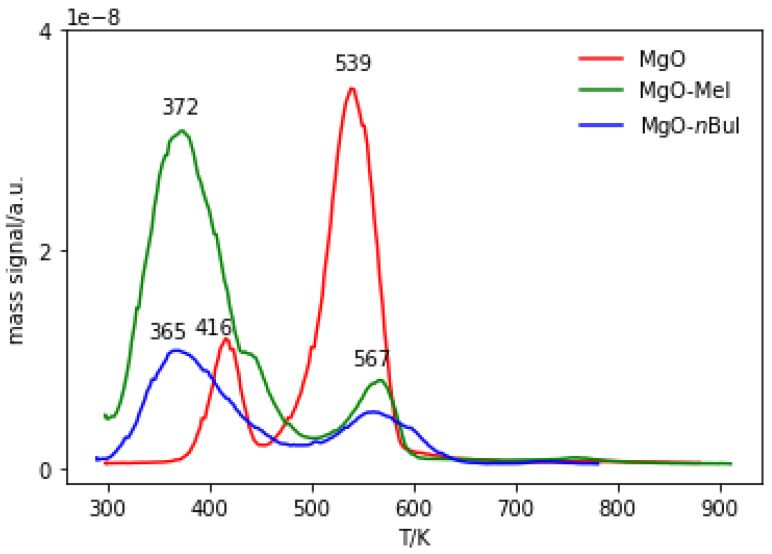
TPD of MeCN from MgO, MgO-MeI, and MgO-*n*-BuI.

**Figure 6:**
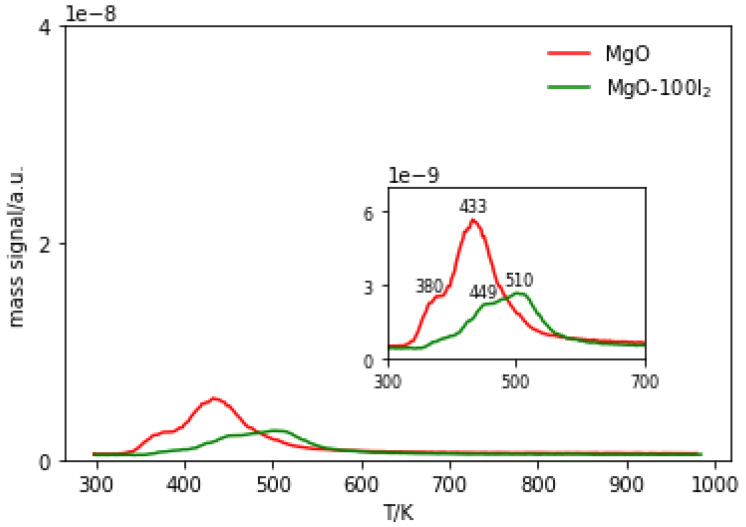
TPD of Et_3_N from MgO and MgO-I_2_ (MeOH).

**Figure 7:**
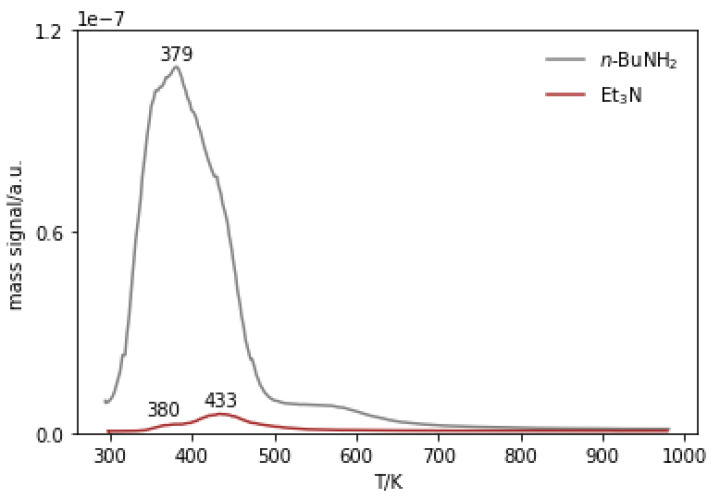
TPD of Et_3_N and *n*-BuNH_2_ from MgO.

**Figure 8:**
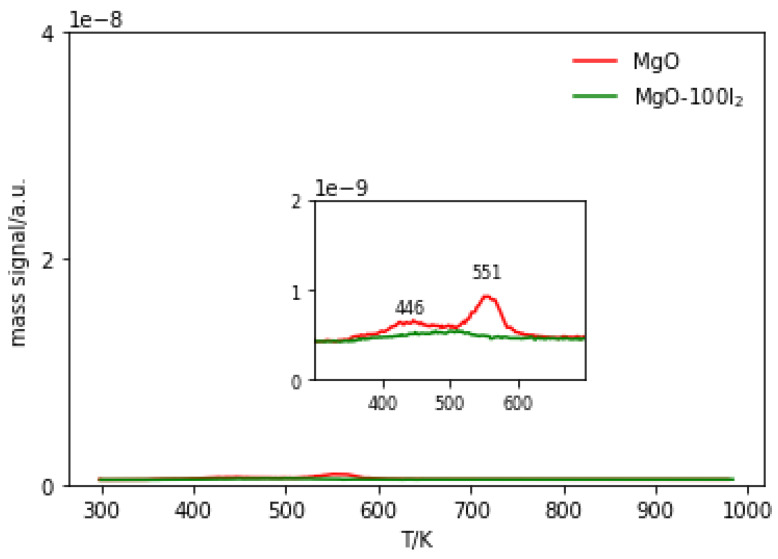
TPD of MeCN (derived from Et_3_N) from MgO and MgO-I_2_ (MeOH).

Four compounds were used as probe molecules for the TPD measurements: acetonitrile (MeCN), pivalonitrile (*t*-BuCN), triethylamine (Et_3_N)), and *n*-butylamine (*n*-BuNH_2_). Both of the nitrile molecules possess a lone electron pair that can react with low coordinated magnesium cations (Lewis acidic sites), but only MeCN can also probe basic sites due to the acidity of the protons of the methyl group caused by the electron-withdrawing properties of the CN group. The acidic strength of MeCN is comparable with that of acetylene, and for both compounds, the pKa value equals 25 [37]. Both nitriles can also exhibit basic properties because they can react with very strong Brønsted acidic sites. However, it does not happen in the case of pure MgO due to very weak Brønsted acidic properties exhibited by the surface of the oxide during the measurements of strength of its acidic sites (Section 2.3.2.). The pK_BH+_ values for *t*-BuCN and MeCN are −10.1 [38] and −10.4 [37], respectively. The pK_BH+_ values for Et_3_N and *n*-BuNH_2_ are 10.75 and 10.64, respectively [37]. The TPD profiles of pure MgO and modified MgO are presented in Figure 2, Figure 3, Figure 4, Figure 5, Figure 6, Figure 7 and Figure 8. 

The sample of pure MgO exhibited two desorption peaks of *t*-BuCN: one at 345 K, which is attributed to the desorption of physisorbed molecules, and a sharp, much larger one, at 527 K. This result indicates that the catalyst surface possesses a substantial number of Lewis acidic sites. For the MgO-I_2_ catalyst, the first peak of pivalonitrile desorption is observed at a much lower temperature, 381 K, which is assigned to the desorption of physisorbed nitrile and/or its interaction with very weak Lewis acidic sites. A second small peak of the nitrile desorption, located on a broad slope, is observed at around 550 K. It can be attributed to the desorption of the nitrile from strong Lewis acidic sites, although present at a low concentration. A similar interpretation can describe the desorption profiles of *t*-BuCN from the surface of MgO-MeI and MgO-*n*-BuI systems. The introduction of modifiers onto the MgO surface results in a very strong reduction in the intensity of peaks (around 550 K), attributed to Lewis acid sites, and the appearance of peaks in the area of 368–371 K, which can be attributed to physisorbed molecules of the nitrile. Low-intensity peaks located around 550 K correspond to the desorption of nitrile from strong Lewis acid sites.

The image of MeCN desorption from pure MgO looks very similar to the desorption of *t*-BuCN, with the difference that the first desorption peak appears at a temperature of only 416 K, i.e., 61 degrees above the boiling point of MeCN. It is related to the desorption of the nitrile molecules from the strongest basic sites of MgO, which are able to deprotonate such a weak C-H acid as MeCN. The second, intensive desorption peak at 539 K is attributed to the desorption of the nitrile molecules from strong Lewis acid sites. In the TPD MeCN profile for the MgO-I_2_ system, the presence of two low-intensity peaks with maximum temperatures of 364 and 558 K was noted. The first peak describes the desorption of physisorbed nitrile, and the second one is attributed to the desorption of nitrile from Lewis acidic sites with an acid strength higher than that observed in the case of pure MgO. A similar interpretation can be used to describe the desorption profiles of MeCN from the surfaces of the MgO-MeI and MgO-*n*-BuI systems (Figure 5). The introduction of modifiers onto the MgO surface causes a significant reduction in the intensity of the peaks assigned to Lewis acidic sites, with a simultaneous increase in their strength indicated by an increase in the maximum temperature from 539 K (for MgO) to 567 K and the appearance of new peaks in the range of 365–372 K, which can be attributed to the physisorbed molecules of the nitrile. 

The very low intensity of the desorption peak of triethylamine at 433 K from the surface of MgO compared to the intensity of the desorption peaks of both nitriles is particularly noteworthy (Figure 6). What is more, a pronounced difference in the intensity of the desorption peaks of two amines, namely Et_3_N and *n*-BuNH_2_ from MgO, was also noted (Figure 7). The primary amine reacts with Brønsted acidic sites, also with Lewis acidic sites, and due to the presence of protons in NH_2_ group, it can react, and indeed, it does with the strongest basic sites classified as superbasic sites present on the surface of pure MgO. What is equally important, both amines show practically the same basicity measured by the value of the pK_BH+_: 10.75 and 10.64 for Et_3_N and *n*-BuNH_2_, respectively [36]. According to literature reports, the size of the bulky tertiary amine hinders the lone electron pair of the nitrogen atom from coming into the proximity of and reacting with a Lewis acidic site. This is why it preferentially reacts with Brønsted acidic sites, i.e., protons, rather than Lewis acidic sites, i.e., low coordinated Mg^2+^ ions. In the case of MgO, previous measurements excluded the presence of Brønsted acidic sites on its surface, hence the peak at 433 K must have a different origin. The behavior of Et_3_N in the case of adsorption on strong Lewis acidic sites is described in the literature [39]. As has been shown, the amine decomposition products of ethylene, hydrogen, and acetonitrile desorb from such sites at elevated temperatures. The authors propose the course of amine decomposition according to the following equations:
(13)
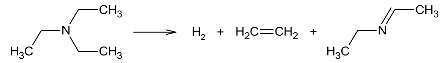

(14)
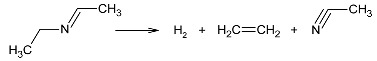


The introduction of I_2_ onto the MgO surface results in the appearance of a peak at 510 K on the low-temperature slope, of which the presence of a low-intensity signal at 449 K can also be observed. The origin of the Et_3_N desorption peaks from both MgO and MgO-I_2_ surfaces was analyzed. For this purpose, the desorption of acetonitrile originating from the decomposition of adsorbed triethylamine was examined because, according to the view prevailing in the literature, nitrile is formed only in the case of decomposition of amine adsorbed on strong acidic Lewis sites (Figure 8). It has been shown that acetonitrile is formed from the decomposition of Et_3_N only on the surface of MgO.

#### 2.3.4. Temperature-Programmed Desorption (TPD) and Mass Spectrometry (MS) of Reaction Products Derived from Methyl Iodide and MgO

**Figure 9:**
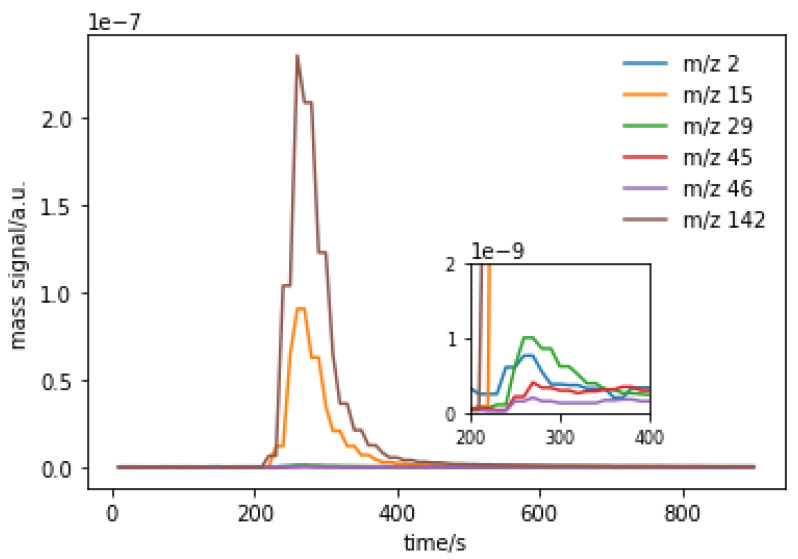
MS of reaction products derived from MeI and MgO. T= const. = 373 K.

The signals for specific m/z values presented in the graphs were selected on the basis of preliminary scanning of the reaction products of methyl iodide with magnesium oxide in the m/z range from 1 to 200. Then, m/z values were selected for analysis, and measurements were performed again. In the reaction of MeI with MgO, data were collected for fragment ions with m/z = 2, 15, 29, 45, 46, and 142 (Figure 9). The most intensive signals are at m/z = 15 and 142. Both come from MeI, part of which did not react and is present in the post-reaction mixture. m/z = 142 is the molecular and fundamental peak for methyl iodide, while the signal m/z = 15 corresponds to the CH_3_^+^ fragment ion. Under the reaction conditions, in addition to the fragmentation of methyl iodide, CH_3_^+^ ion may be formed as a result of the decomposition of methane and dimethyl ether. Fragmentation of the methane molecule leads to the formation of ions with m/z = 15 and 16. The latter signal was not analyzed during detailed studies, but its presence was detected during scanning in the range of m/z values from 1 to 200, which confirms the formation of this compound. The presence of dimethyl ether in the post-reaction mixture was confirmed based on the presence of signals with m/z = 15, 29, 45, and 46 in the MS spectrum. Its formation can be described by the following equations:

(15) $${\left[MgO\right]}_{\left(surf\right)}+{CH}_{3}I_{\left(gas\right)}\to {\left[MgI\left(O{CH}_{3}\right)\right]}_{\left(surf\right)}$$

(16) $${\left[MgI\left(O{CH}_{3}\right)\right]}_{\left(surf\right)}+{CH}_{3}I_{\left(gas\right)}\to {\left[MgI_{2}\right]}_{\left(surf\right)}+H_{3}C-O-CH_{3}$$

**Figure 10:**
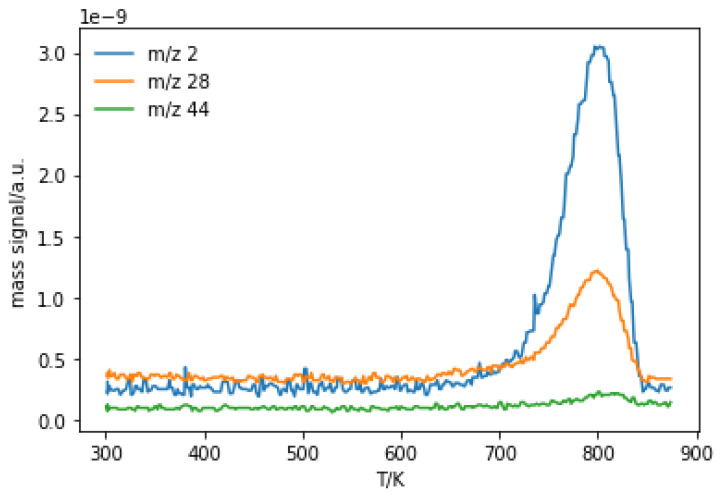
TPD-MS of reaction products derived from MeI and MgO.

The signal with m/z = 29 could come from formaldehyde, but no signal with m/z = 30 was found in either preliminary or detailed studies, the presence of which would confirm the formation of this compound. Similarly, the presence of methanol, ethane, and ethylene was not detected. The absence of ethane in the reaction products indicates that the reaction does not proceed via the radical reaction pathway [40]. The TPD-MS measurement results are shown in Figure 10. The organic groups remaining on the MgO surface (e.g., OCH_3_) decompose at higher temperatures, which results in the desorption of hydrogen (m/z = 2), carbon monoxide (m/z = 28), and very small amounts of carbon dioxide (m/z = 44) at a temperature of about 800 K. The hydrogen desorption signal is the most intensive. The results obtained in the reaction of EtI and *n*-BuI with MgO analyzed using both methods (MS and TPD-MS) were not included in the work due to their complexity and difficulties encountered during the interpretation of the obtained data. 

#### 2.3.5. Fourier Transform Infrared Spectroscopy (FTIR)

**Figure 11:**
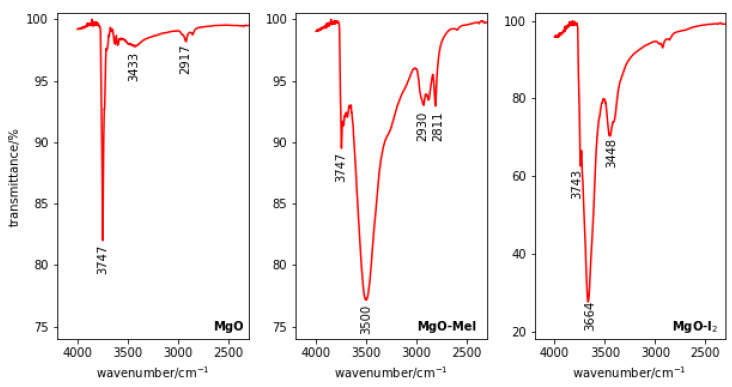
FTIR spectra of MgO, MgO-MeI, and MgO-I_2_.

The FTIR spectra of MgO, MgO-MeI, and MgO-I_2_ recorded in the range of wave numbers from 4000 to 2500 cm^−1^ are depicted in Figure 11. The band at 3747 cm^−1^ was assigned to isolated hydroxyl groups and low-intensity bands in the range of 3000–2800 cm^−1^ are most likely related to the presence of vacuum grease (apiezon). In part of the spectrum of MgO (not shown in Figure 11), the presence of bands in the area of 1700–1200 cm^−1^ was also found, which were attributed to magnesium carbonates. After the reaction of MeI or I_2_ with MgO, the intensity of the band originating from free hydroxyl groups (3747 cm^−1^) decreased significantly. However, new, very intense bands appeared at 3500 and 3664 cm^−1^ for MgO-MeI and MgO-I_2_, respectively. In the spectrum of the former, one can observe multiple new bands related to the stretching vibrations of C-H groups derived from the modifier. Based on the literature data, the band around 2930 cm^−1^ can be assigned to methyl iodide. The complexity of the bands in the spectrum ranging from 3660 to approximately 3300 cm^−1^ indicates the presence of various types of hydroxyl groups formed on the MgO surface after its modification with MeI or I_2_.

### 2.4. Catalytic Activity of MgO-X_2_, MgO-HX, and MgO-RX Systems in Transfer Hydrogenation Reaction between Ethanol and Acrolein

Studies on catalytic hydrogen transfer from ethanol, as the hydrogen donor, to acrolein, as the acceptor, in the presence of selected catalytic systems were performed. The hydrogen donor was ethanol, because, as we showed earlier, secondary alcohols show lower reactivity in this reaction than that determined via thermodynamic calculations [41]. Measurements of catalytic activity were carried out in the temperature range of 473–573 K, i.e., in the range in which the highest yield of allyl alcohol was recorded for other catalytic systems. Transfer hydrogenation from ethanol to acrolein leads to the formation of three products derived from acrolein, allyl alcohol (UOL), propanal (SAL), and 1-propanol (SOL), according to the following equation:
(17)
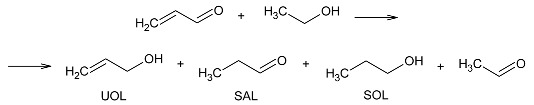


During the reaction, several by-products can form. Acetaldehyde, which is a product of dehydrogenation of ethanol, can react via Equation (18) to form 1,1-diethoxyethane, or via Equation (19) to form crotonaldehyde (2-butenal) formed in the aldol condensation reaction of acetaldehyde. Acrolein can react with more than one molecule of the hydrogen donor to form products such as 1,1-diethoxy-2-propene, Equation (20), and 1,1,3-triethoxypropane, Equation (21). The latter product is formed as a result of two reactions: Michael addition of ethanol to acrolein with the formation of 3-ethoxypropanal and its subsequent acetalization. Brønsted acidic sites, necessary for the acetalization reaction, are formed on the surface of MgO after the reaction of the oxide with some modifiers. The presence of all these compounds in the post-reaction mixtures was evidenced using GC-MS.
(18)
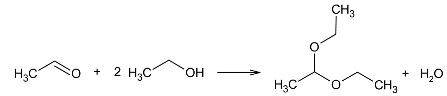

(19)
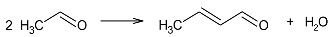

(20)
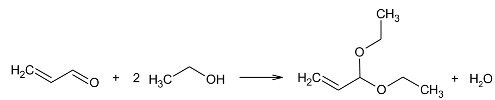

(21)
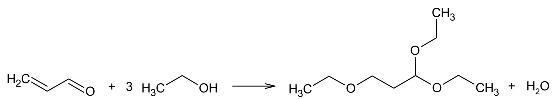


Several alcohols were used for the synthesis of the modified MgO catalysts. The synthesis of iodine and bromine-modified catalysts was carried out using methanol as a reagent and solvent via wet impregnation. It was shown that modification with methanol alone had no effect on the activity of MgO in the studied reaction. The results of the transfer hydrogenation reaction tests of the MgO-X_2_ catalysts, where X = I or Br ([X_2_] = 0–300 μmol∙g^−1^), obtained using methanol, are presented in Table 13 and Table 14, respectively. The introduction of iodine as an MgO modifier resulted in a strong increase in the catalytic activity of the oxide, as determined by the change in the conversion of acrolein, from 15 to 45% at a temperature of 473 K (Table 13), already for the lowest modifier concentration of 50 μmol∙g^−1^. Moreover, the high chemoselectivity (ChS) of the reaction towards the formation of allyl alcohol (UOL) of pure MgO is maintained (100%). At the highest reaction temperature (573 K), conversions of 51 and 76% were recorded for MgO and MgO-I_2_, respectively. A further increase in the modifier concentration (100 and 200 μmol∙g^−1^) on the surface of the MgO-I_2_ catalyst causes a slight increase in conversion, up to 82%. The chemoselectivity to UOL at this temperature decreases with the increase in the modifier concentration. The concentrations of the modifier (μmol∙g^−1^) and ChS (%) are as follows: 0/92 > 50/91 > 100/82 > 200/57. Only minute amounts of propanal (SAL) (1–2%) were observed in the post-reaction mixtures. The presence of another reaction product, 1-propanol (SOL), was also determined; its yield increased with the reaction temperature and amounted to a maximum of 10%. In the case of the MgO-200 I_2_ catalyst, the significant yields of products classified as “others” are noteworthy. These are condensation and aldolization products. The reactions leading to their formation occur due to the presence of Brønsted acidic sites on the surface of MgO, which are known to catalyze this type of transformation. The use of ethanol, instead of methanol, as a reagent and solvent in the synthesis of the MgO-100 I_2_ system results in a catalyst with identical activity to the one obtained using methanol, but with a lower chemoselectivity.

A similar trend of activity in the transfer hydrogenation of acrolein was observed for the MgO-Br_2_ catalysts (Table 14). At the lowest reaction temperature, 473 K, 100% of the chemoselectivity towards allyl alcohol was noted at 63 and 72% conversion of acrolein for MgO-100 Br_2_ and MgO-200 Br_2_, respectively. An increase in the reaction temperature to 523 K resulted in a further increase in the conversion and UOL yield for these catalysts, with a simultaneous decrease in chemoselectivity. The MgO-300 Br_2_ system was characterized by similar activity as in the case of MgO-100 Br_2_, but with significantly lower chemoselectivity to UOL (only 50% at 523 K), yielding by-products classified as “others” of up to 38%.

The high catalytic activity of MgO-X_2_ systems in transfer hydrogenation with the observed very high chemoselectivity towards the formation of UOL prompted us to synthesize MgO-HX catalysts (X = Cl, Br or I, [HX] = 200 μmol g^−1^) with the same concentration of halide anions as in the case of MgO-X_2_ ([X_2_] = 100 μmol g^−1^). The results of the measurements of their catalytic activity in the studied reaction are compiled in Table 15. It has been shown that the introduction of halide ions at a concentration of 200 μmol g^−1^ to the MgO surface causes a significant increase in the activity of such a system compared to unmodified MgO, and this increase depends on the type of halide ion. At a temperature of 523 K, the activity of MgO-HX systems, measured by the conversion of acrolein, changes as follows: 73% (HI) > 69% (HBr) > 56% (HCl). A high chemoselectivity towards UOL was observed and decreased with temperature. At the lowest temperature (473 K), the chemoselectivity was 100% (MgO-HCl), and it was 98% for the other two studied systems. At 573 K, these values were 89% for MgO-HCl, 95% for MgO-HBr, and 91% for MgO-HI. As shown in an independent test using H_3_PO_4_ as an MgO modifier, the type of anion applied onto the MgO surface has a decisive influence on the activity of the new system created as a result of the modification. It was noted that the MgO-H_3_PO_4_ system faithfully reproduces the activity profile of pure MgO in the reaction. 

The last part of this research was the measurement of the activity of MgO-RX series catalysts. Methyl iodide and EtX, where X = Cl, Br, or I, were selected as magnesium oxide modifiers. Each alkyl halide led to an increase in the activity of the formed catalyst in comparison to pure MgO (Table 16). The extent of the increase depended on the type of modifier. In the case of the MgO-MeI system, the highest UOL yield (65%) was achieved already at a temperature of 473 K. An increase in the reaction temperature resulted in an increase in the yield of 1-propanol (SOL) of up to 21% at the expense of UOL. The MgO-EtCl system was the least active catalyst for the transformation of acrolein, although at a temperature of 473 K, in its presence, a chemoselectivity to UOL of 100% was achieved at a conversion of 45%. However, at a temperature of 573 K, by-products were formed in its presence with a yield of 38%. The MgO-EtBr and MgO-EtI systems were characterized by a higher activity than that observed in the case of the MgO-EtCl system. Moreover, with UOL yields of 70–77%, the chemoselectivity of its formation was 96–97% in the temperature range of 473–523 K.

## 3. Materials and Methods

### 3.1. Solvents and Organic and Inorganic Reagents

Triple-distilled water was used in the experiments performed in this work. Sulfuric acid (p.a., 98%, POCH, Gliwice, Poland) was used as received. Starch (analytical reagent, soluble, BDH, London, UK) was used as a water solution, stabilized by the addition of a small amount of salicylic acid. Bromine (>99.5%, Aldrich, Poznań, Poland), iodine (p.a., resublimed), iodine bromide, and iodine trichloride (both from BDH, London, UK) were used as received. Hydrochloric acid (38%, p.a., POCH Gliwice, Poland), hydrobromic acid (48%, ACS reagent, Aldrich, Poznań, Poland), and hydroiodic acid (57%, no stabilizer, Aldrich, Poznań, Poland) were used as received. The concentrations of hydrohalic acids were verified using the titration method, with 0.1 M KOH in the presence of phenolphthalein used as an indicator.

Chloroform (pure, POCH, Gliwice, Poland) was washed three times with distilled water to remove ethanol and dried at 273 K under nitrogen over anhydrous CaCl_2_. Next, it was distilled in a stream of dry nitrogen in an all-glass apparatus. The distillate was kept under N_2_ in a Schlenk-type container covered with metallic foil. 

Alcohols: Methanol (p.a., POCH, Gliwice, Poland) and ethanol (p.a., anhydrous 99.8%, POCH, Gliwice, Poland) were dried according to the Lund–Bjerrum method using metallic magnesium and iodine. Commercial alcohols: propan-2-ol (p.a., POCH, Gliwice, Poland), pentan-2-ol (98%, Aldrich, Poznań, Poland), pentan-3-ol (98%, Aldrich, Poznań, Poland), and 2-methylpropan-2-ol (*t*-butanol, 99%, Aldrich, Poznań, Poland) were distilled over metallic potassium in nitrogen and kept dry in Schlenk-type containers. 

Toluene (p.a., POCH, Gliwice, Poland) was pre-dried using fractional distillation under normal pressure. The first 10% of the distillate was discarded. The final drying was performed using metallic sodium and benzophenone in a nitrogen atmosphere. The anhydrous distillate was collected in a Schlenk-type container under nitrogen.

Alkyl halides: The following compounds were used:

alkyl chlorides: MeCl, CH_2_Cl_2_, CHCl_3_, CCl_4_, *n*-BuCl, *i*-BuCl, *s*-BuCl, and *t*-BuCl;
alkyl bromides: MeBr, CH_2_Br_2_, CHBr_3_, *n*-BuBr, *i*-BuBr, *s*-BuBr, and *t*-BuBr;
alkyl iodides: MeI, CH_2_I_2_, CHI_3_, EtI, *n*-BuI, *i*-BuI, *s*-BuI, and *t*-BuI;
other alkyl halides: BrCH_2_Cl, ClCH_2_I, BrCH_2_I, and BrCH_2_CH_2_CH_2_Cl.

Most of the halides are commercial products from Aldrich (Poznań, Poland) and Fluka Chemie GmbH (Buchs, Switzerland). They were purified via a standard treatment consisting of washing with a 5% NaHCO_3_ solution, with distilled water, drying over anhydrous CaCl_2_, and fractional distillation under normal or reduced pressure (CHBr_3_, CH_2_I_2_ and *t*-BuI). Prior to this treatment, alkyl iodides were washed with a solution of sodium thiosulphate to remove traces of iodine. Ampoules containing methyl chloride and methyl bromide, both from BDH (London, UK), were cooled in dry ice and opened, and the halides were dried over cooled anhydrous CaCl_2_ at 240 K. CHI_3_ (99%) from Aldrich (Poznań, Poland) was crystallized twice from ethanol. CH_2_BrI was synthesized from CH_2_Br_2_ and NaI in boiling acetone. The product was purified via rectification under normal pressure; b.p. 412–413 K/1001 hPa (exp.), 411–414 K/1013 hPa (lit.); yield, 11%.

Acrolein (90%, Aldrich, Poznań, Poland) was dried over anhydrous MgSO_4_ at 273 K and distilled under normal pressure in nitrogen. The fraction with the boiling point of 325–326 K was collected. This fraction was treated in the same manner as above. The distillate (b.p. 325.5–326.0 K) was collected in a Schlenk-type container and kept at 243 K in a freezer. The final purity was 99.4% (GC).

Acetonitrile (99.8%, anhydrous, Aldrich, Poznań, Poland) and pivalonitrile (98%, Aldrich, Poznań, Poland) were dried over P_2_O_5_ and distilled in a nitrogen atmosphere under normal pressure with the addition of a small amount of fresh P_2_O_5_. Triethylamine (98%, Aldrich, Poznań, Poland) and *n*-butylamine (99%, Aldrich, Poznań, Poland) were dried over KOH pellets for 2 weeks and distilled under normal pressure in a nitrogen atmosphere in the presence of metallic potassium. 

Potassium iodate, KIO_3_ (p.a., Aldrich, Poznań, Poland) was dried in an oven at 423 K for 4 h and kept in a tightly closed container. Potassium iodide, KI (p.a., POCH, Gliwice, Poland) and sodium thiosulphate pentahydrate, Na_2_S_2_O_3_·5H_2_O (99.5%, Aldrich, Poznań, Poland) were used as received. A stock solution (~0.1 M) of Na_2_S_2_O_3_ was prepared and stabilized via the addition of a small amount of CHCl_3_. Sodium thiosulphate solutions with lower concentrations (0.001–0.005 M) were prepared by diluting the stock solution. Their concentrations were determined after a 24 h period using anhydrous KIO_3_ as a standard in the presence of iodide ions in acidic solution.

### 3.2. Synthesis of Magnesium Oxide

Analytically pure magnesium oxide (purum p.a., Reachim, Chişinâu, Moldova) was subjected to an additional purification step, which has been described before [28]. In brief, it was digested in nitric acid and precipitated with ammonia water (25% solution, POCH, Gliwice, Poland) in stages, with the first precipitate discarded. The powder of purified Mg(OH)_2_ was pelletized, and the pellets were crushed. A sieved fraction of 0.16–0.40 mm was calcined in a tubular quartz reactor at 873 K for 1 h in a stream of air and for 5 h in a stream of dry deoxygenated nitrogen. After cooling in a stream of nitrogen, the oxide was transferred to a Schlenk-type container and stored under nitrogen.

### 3.3. Reaction of MgO with Br_2_, I_2_, or HX in Alcohols

Three procedures for the introduction of bromine or iodine onto the surface of the oxide were used. For each procedure, all operations connected with the sample preparation and handle were performed in a dry nitrogen atmosphere.

In the first procedure, a sample of MgO in alcohol was treated with solid iodine. The solid iodine was added in one portion to a previously prepared suspension of the oxide, which consisted of approx. 200 mg of MgO (0.04–0.16 mm grain diameter) and 20 cm^3^ of an anhydrous alcohol (methanol, ethanol, propan-2-ol, pentan-2-ol, pentan-3-ol, or 2-methylpropan-2-ol), which was placed under dry nitrogen in a Schlenk-type container equipped with a stirring bar and a double-jacketed glass condenser. The molar ratio pf I_2_/MgO was 0.5. The mixture was heated to reflux for 6 h. After cooling, the condenser was removed, and a glass set for distillation with a trap cooled with dry ice and a receiver was installed. A slow distillation was performed under reduced pressure (1.5–2.0 kPa). The concentrations of I^−^ ions in the solid residue were measured. The presence of IO_3_^−^ ions was also checked.

In the second procedure, solid iodine (200 μmol g^−1^ or 3.00 mmol g^−1^ of MgO) or a solution of bromine in methanol (200 μmol g^−1^ or 3.00 mmol g^−1^ of MgO) was added to a suspension of MgO (200 mg) in anhydrous methanol (10 cm^3^). The mixture was kept in the dark for the appropriate period of time at an ambient temperature with occasional shaking. The same procedure was used for the preparation of MgO-X_2_ (X = Br or I) and MgO-HX (X = Cl, Br or I) catalysts containing 100, 150, or 300 μmol of X_2_ or 200 μmol of HX per 1 g of MgO. A solution of a halogen/hydrohalic acid in methanol was added to a suspension of the oxide in methanol under nitrogen. The reaction was performed in the darkness for 24 h in order to ensure a quantitative conversion of the halogen. An excess of liquid was distilled off under reduced pressure, and the solid product was calcined at 373 K in a stream of pure nitrogen.

In the third procedure, a stream of deoxygenated anhydrous nitrogen (20 cm^3^ min^−1^) was passed through a glass saturator filled with bromine kept at 273 K. The stream of N_2_ saturated with halogen vapors was passed through a fixed MgO bed placed in a tubular quartz reactor and heated to the appropriate temperature. Usually, after 3 h of contact with the vapors, the bed of metal oxide was purged with pure nitrogen at the same temperature for 1 h and cooled in a stream of N_2_.

### 3.4. Reaction of MgO with RX, IBr, or ICl_3_ Vapors (with the Exception of MeCl, MeBr, and CHI_3_)

A stream of pure, anhydrous nitrogen (20 cm^3^ min^−1^) was passed through a glass saturator filled with the appropriate alkyl halide at 293 K (373 K for interhalogen compounds). Then, the stream of N_2_ saturated with vapors was passed through a magnesium oxide bed (600 mg) kept in a tubular quartz reactor and heated to the appropriate temperature (373 ± 2 K). After 3 h of contact with the vapors, the bed of oxide was purged with pure nitrogen at the same temperature for 1 h and cooled in N_2_.

### 3.5. Reaction of MgO with MeCl or MeBr Vapors

The same glass apparatus was used as above. Anhydrous CaCl_2_ was placed in the saturator. Cold (230 K) liquid alkyl halide was poured onto the calcium chloride. The temperature of the saturator was kept in the range of 230–240 or 250–260 K for methyl chloride and methyl bromide, respectively. After 3 h of contact with MgO, kept at 373 ± 2 K, the bed of oxide was purged with pure nitrogen at the same temperature for 1 h and cooled in N_2_.

### 3.6. Reaction of MgO with CHI_3_ Vapors

Due to the low volatility of CHI_3_ and its susceptibility to decomposition around its melting point (395–396 K), a modified version of the reactor was used. A fixed bed of MgO was placed in the reactor. Above the bed, there was a special adapter with a glass basket into which the CHI_3_ was loaded (~100 mg). The basket was lowered into the reactor and kept slightly above the oxide bed. A stream of N_2_ was introduced downwards to the reactor, which was kept at 373 ± 2 K using external electric heating. The evaporation of CHI_3_ was performed until the whole bed of MgO turned yellow (10 h). Next, the oxide was purged with pure nitrogen at the same temperature for 3 h and cooled in N_2_.

### 3.7. Analytical Determinations

#### 3.7.1. Qualitative Tests for I^−^ and IO_3_^−^ Ions

Excess iodine from a sample of MgO reacted previously with a solution of iodine was extracted using chloroform (5 times). Next, the remaining solvent was removed from the sample under reduced pressure. The resulting solid was digested using 1 M H_2_SO_4_. The obtained solution was treated either with 30% H_2_O_2_ (p.a., POCH, Gliwice, Poland) or with a freshly prepared 0.1 M KI solution in the presence of starch for the confirmation of the presence of I^−^ and IO_3_^−^ ions, respectively.

#### 3.7.2. Quantitative Test for I_2_ on MgO

All operations were performed in a dry nitrogen atmosphere. A weighed sample of the modified oxide (250 mg) was placed in an Erlenmeyer flask (100 cm^3^) fitted with a ground-glass stopper. After the addition of 15 cm^3^ of 1 M H_2_SO_4_, the mixture was stirred for 5 min. Next, the free iodine was extracted with CHCl_3_ (5 × 3 cm^3^) in a separatory funnel. The organic extracts were collected, washed with water, and treated with a solution of 5 cm^3^ 0.1 M KI in 5 cm^3^ of water. The two-phase mixture was titrated with 0.002 M Na_2_S_2_O_3_ in the presence of starch.

#### 3.7.3. Quantitative Test for Br_2_ on MgO

All operations were performed in a dry nitrogen atmosphere. A weighed sample of modified oxide (250 mg) was placed in an Erlenmeyer flask (100 cm^3^) fitted with a ground-glass stopper. After the addition of 15 cm^3^ of 1 M H_2_SO_4_, the mixture was stirred for 5 min. An excess of 0.1 M KI solution was added. Free iodine was extracted using CHCl_3_ (5 × 3 cm^3^) in a separatory funnel. The organic extracts were collected, washed with water, and treated with a solution of 5 cm^3^ of 0.1 M KI in 5 cm^3^ of water. A two-phase mixture was titrated with 0.002 M Na_2_S_2_O_3_ in the presence of a starch.

#### 3.7.4. Quantitative Test for I^−^ Ions on MgO

In order to determine the concentration of iodide ions, the acidic water layer left after the determination of I_2_ was combined with 1 cm^3^ of 0.005 M NaIO_3_, and the liberated iodine was extracted using chloroform (5 × 3 cm^3^). The extracts were collected, washed with water, and titrated using 0.002 M Na_2_S_2_O_3_.

#### 3.7.5. Quantitative Test for Cl^−^ and Br^−^ Ions on MgO

A sample of MgO-X (approx. 200 mg) was digested in a small volume of 20% nitric acid, diluted to 10.0 cm^3^ with distilled water, and placed in a 150 cm^3^ vessel. Next, 5.0 cm^3^ of 0.2 M HNO_3_ and 5.0 cm^3^ of 2.0 M KNO_3_ were added, and the resulting solution was diluted to 100.0 cm^3^ with distilled water. The mixture was then stirred and titrated with a 0.01047 M solution of AgNO_3_ using an automatic titrator with an Ag indicator electrode and an Ag-AgCl double-junction reference electrode. The titration curve was recorded, and the titrant volume at each selected potential point (in 2 mV interval) was collected. To improve the precision of the determination, the measurements were repeated three times, and the average values of the halide contents were calculated. The titrations were carried out using a model Metrohm 702 Titrino automatic potentiometric titrator (Metrohm AG, Herisau, Switzerland). An Ag indicator electrode (Orion) was used as the working electrode, and a double-junction Ag-AgCl electrode (Orion) was used as the reference electrode (inner chamber filling with a solution saturated with AgCl and outer chamber filling with 20% KNO_3_ solution). All the reagents used were of analytical reagent grade.

#### 3.7.6. Analysis of Organic Reaction Products

After completing the reaction of I_2_ with MgO in the presence of alcohols, the liquid products were distilled off under reduced pressure and condensed in a trap cooled using a dry ice-propan-2-ol mixture. The reaction products were analyzed using GC using HRGC 4000B KONIK (Barcelona, Spain) equipped with a TRACER WAX capillary column (length 30 m, 0.25 mm i.d., 0.25 μm film thickness) and an FID detector. The compounds were identified using GC-MS (HP-6890N with a 5973N mass detector) (Agilent, Santa Clara, CA, USA). In the case of the MgO-MeI catalyst, the reaction of methyl iodide with MgO was monitored using mass spectrometry. For this purpose, 100 mg of MgO was placed in a quartz flow reactor and calcined in a He flow (25 cm^3^ min^−1^) for 1 h at 873 K. After cooling to a temperature of 373 K, the He flow was reduced (10 cm^3^ min^−1^), and 5 · 10^−3^ cm^3^ of the appropriate liquid modifier was administered three times, analyzing the products formed as a result of the reaction using a mass spectrometer. After cooling the catalyst sample to 298 K, the TPD-MS measurement was performed by heating the sample to a temperature of 873 K with a heating ramp of 10 deg min^−1^. The desorption products were analyzed using a Dycor Ametek MA200 quadrupole mass spectrometer (Pittsburgh, PA, USA) in the m/z value range of 1 to 200.

### 3.8. Characterization of MgO, MgO-X_2_ (X = Br and I), MgO-HX, and MgO-RX Catalysts

The purified magnesium oxide, its precursor—Mg(OH)_2_, and the modified catalysts were characterized using a number of techniques. The surface areas of the samples were measured using ASAP2020 (Micromeritics Instrument Co., Norcross, GA, USA). Before the measurement, the samples were outgassed for 3 h at 373 K. The total specific surface area (S_BET_) was determined using the Brunauer–Emmett–Teller adsorption isotherm model in the relative pressure range of 0.05–0.3. Powder diffraction data were collected using a D-5000 diffractometer (Bruker AXS, Karlsruhe, Germany) equipped with a scintillation counter and Ni-filtered Cu K_α_ radiation. Before the measurements, the samples were immobilized in anhydrous silicon grease. The TG-DTA measurements were performed using an STA 449C thermobalance (NETZSCH, Selb, Germany). The samples were heated to 873 K under an Ar flow (10 cm^3^·min^−1^, heating ramp 10 deg∙min^−1^). The data were processed using NETZSCH Proteus Thermal Analysis software (version 6.1.0). The strength of the surface acid–base sites of the catalysts was determined using the Hammett method using a sequence of indicators in anhydrous toluene as the solvent [36]. The following set of indicators was used (the values of pK_A_ and pK_BH+_ are given in parentheses): chalcone (−5.6), dicinnamylideneacetone (−3.0), crystal violet (0.8), methyl red (4.8), bromothymol blue (7.2), phenolphthalein (9.3), 2,4-dintroaniline (15.0), 4-nitroaniline (18.4), diphenylamine (22.3), 4-chloroaniline (26.5), and triphenylmethane (33.0). The measurements were performed under dry nitrogen at room temperature with reading after 24 h.

IR spectroscopy: Measurements were carried out using a Thermo Scientific NICOLET 6700 FTIR spectrometer (London, UK) with an MCT detector. A fine powder of MgO, freshly calcined at 873 K for 5 h, was pressed into a thin wafer using a steel die (20 mm i.d.) under a pressure of 30 MPa. The wafer was placed in a quartz holder, which was suspended in a set consisting of an electric tube furnace connected to an IR cell equipped with CaF_2_ windows and a vacuum system. It was calcined at 873 K for 1 h in air and at the same temperature for 3 h under a pressure of 6 Pa. After cooling the wafer to room temperature, its spectrum was recorded. The following measurements were performed, and the spectra were recorded:

- MeI adsorption on MgO at 373 K for 10 min, desorption at 293 K for 10 min;
- I_2_ adsorption on MgO at 473 K for 15 min; desorption at 473 K for 15 min.

### 3.9. Catalytic Activity Measurements of MgO-X (X = Cl, Br and I) Catalysts in Transfer Hydrogenation of Acrolein with Alcohols

Catalytic activity measurements were carried out using a fixed-bed tubular glass reactor into which a sample of the catalyst (0.250 ± 0.005 g) was loaded in a stream of dry nitrogen. A solution of acrolein in a hydrogen donor (at a given molar ratio) with *t*-butylbenzene added as an internal standard was dosed using a microdosing pump with a LHSV (liquid hourly space velocity) of 3 h^−1^ into a stream of dry nitrogen (5N, Multax, Stare Babice, Poland) (50 cm^3^∙min^−1^), which was passed through the catalyst bed. The reaction products were collected in glass receivers, cooled to 213–223 K with a propan-2-ol–dry ice mixture. Prior to the activity measurements, the catalyst was maintained at 473 K in the stream of reactants for 60 min to ensure that the result was not influenced by an initially short-lived high activity of the catalyst that would obscure the evaluation of its long-term catalytic properties. Therefore, the activity was evaluated using the reaction mixture taken in the range of 60–90 min. 

The reaction products were analyzed using GC using HRGC KONIK (Barcelona, Spain) equipped with a TRACER WAX capillary column (length 30 m, 0.25 mm i.d.) and a flame ionization detector. The compounds were identified using GC-MS (HP-6890N with a 5973N mass detector) (Agilent, Santa Clara, CA, USA) and based on a comparison of the retention time with that of a standard sample.

## 4. Conclusions

A series of new MgO-based catalysts has been synthesized and tested in catalytic transfer hydrogenation from ethanol to acrolein. All of the modified systems exhibited a higher activity compared to pure magnesium oxide, and the chemoselectivity of the reaction towards the formation of allyl alcohol remained very high for most of these systems. Moreover, the potential of the magnesium oxide to neutralize alkyl halides as model molecules of chlorinated wastes was determined through a systematic study with a wide range of compounds. As a result of extensive research on the reaction of the oxide with 30 alkyl halides, it was found that it is a reactive neutralizer of chlorinated wastes. The highest efficiency of its operation was recorded in the case of secondary and tertiary alkyl halides, for which the reaction took place in the entire volume of MgO. In the reaction of MgO with various modifiers, such as bromine and iodine, interhalogen compounds, hydrohalic acids, and alkyl halides, a new type of catalysts was prepared. New phases, observed using PXRD, were formed only after the modification of MgO with secondary and tertiary alkyl halides. The TPD of the probe molecules (acetonitrile, pivalonitrile, triethylamine, and *n*-butylamine) from the surface of the modified catalysts showed that the concentration of primary Lewis acid sites present on its surface was significantly reduced, with a simultaneous appearance of secondary Lewis acid sites in low concentrations, with a strength slightly higher than that of primary ones. The modification of the MgO resulted in the suppression of the strong basic sites of the oxide, with a simultaneous appearance of Brønsted acidic sites on its surface. Out of the several alcohols used for the introduction of iodine onto the MgO surface, methanol was the best choice, because it led to a complete incorporation of the iodine into the sample. 

## Figures and Tables

**Table 1:** Reaction of Br_2_ vapors with magnesium oxide heated to T_R_ for 3 h.

T_R_ [K]	Concentration [μmol g^−1^ MgO]	
	Br_2_	Br^−^
295	43	125
373	5	120
473	0	115
673	0	117
873	0	115

**Table 2:** Reaction of I_2_ with MgO in various alcohols at their boiling points (reflux for 6 h). Molar ratio I_2_/MgO = 0.5.

Alcohol	B.p. [K]	[I^−^] [mmol g^−1^] ^1,2,3^	([I^−^]/[Mg^2+^]) ^4^_surf_	([I^−^]/[Mg^2+^]) ^5^_bulk_
CH_3_OH	337.9	10.8	5.8	0.44
C_2_H_5_OH	351.5	10.4	5.6	0.42
2-C_3_H_7_OH	355.7	6.8	3.6	0.27
2-C_5_H_11_OH	392.5	12.1	6.5	0.49
3-C_5_H_11_OH	388.5	9.9	5.3	0.40
*t*-C_4_H_9_OH	355.7	6.5	3.5	0.26

^1^ per 1 g of MgO; ^2^ in all cases, an absence of IO_3_^−^ ions was noted; ^3^ [I^−^]_max_ = 24.8 mmol g^−1^ of MgO; ^4^ molar ratio calculated for Mg^2+^ surface cations only; ^5^ molar ratio calculated for all Mg^2+^ cations.

**Table 3:** Main reaction products of I_2_ with MgO in various alcohols at their boiling points (reflux for 6 h). Molar ratio I_2_/MgO = 0.5.

Alcohol	Main Reaction Products
MeOH	No organic reaction products were detected
EtOH	1,1-diethoxyethane, acetaldehyde, ethyl acetate
2-PrOH	2-iodopropane, acetone, di *i*-propyl ether
2-PeOH	2-pentanone, 2-iodopentane, di 2-pentyl ether
3-PeOH	3-pentanone, 3-iodopentane, di 3-pentyl ether
*t*-BuOH	methylpropene, 2,4,4-trimethyl-1-pentene *t*-butyl *i*-butyl ether

**Table 4:** Progress of reaction of I_2_ with MgO suspended in methanol at 295 K for two initial concentrations of I_2_ (200 μmol g^−1^ and 3.00 mmol g^−1^ of MgO).

Time [h]	[I_2_] [μmol g^−1^]	[I^−^] ^1^ [μmol g^−1^]	[I_2_] [mmol g^−1^]	[I^−^] ^2^ [mmol g^−1^]
0.0	200	0	3.00	0.00
0.5	98	211	2.64	0.66
1.0	73	263	2.60	0.69
3.0	41	331	2.57	0.85
6.0	23	368	2.47	1.08
10.0	0	404	2.42	1.13
16.0	0	397	2.37	1.24
24.0	0	402	2.34	1.32
30.0	--	--	2.32	1.34 ^3^

^1^ [I^−^]_max_ = 400 μmol g^−1^ of MgO; ^2^ [I^−^]_max_ = 6.00 mmol g^−1^ of MgO; ^3^ [I_2_] = 2.31 mmol g^−1^ and [I^−^] = 1.35 mmol g^−1^ after 40 h.

**Table 5:** Progress of reaction of Br_2_ with MgO suspended in methanol at 295 K for two initial concentrations of Br_2_ (200 μmol g^−1^ and 3.00 mmol g^−1^ of MgO).

Time [h]	[Br_2_] [μmol g^−1^]	[Br^−^] ^1^ [μmol g^−1^]	[Br_2_] [mmol g^−1^]	[Br^−^] ^2^ [mmol g^−1^]
0.0	200	0	3.00	0.00
0.2	65	270	--	--
0.5	5	382	2.51	0.88
1.0	0	394	2.30	1.36
3.0	0	392	2.01	1.94
6.0	0	395	1.47	3.00
16.0	--	--	0.34	5.25
24.0	--	--	0.06	5.80
30.0	--	--	0.02	5.94 ^3^

^1^ [Br^−^]_max_ = 400 μmol g^−1^ MgO; ^2^ [Br^−^]_max_ = 6.00 mmol g^−1^ MgO; ^3^ [Br_2_] = 0.00 mmol g^−1^ and [Br^−^] = 5.97 mmol g^−1^ after 40 h.

**Table 6:** Properties of MgO after its reaction with various alkyl chlorides. T = 373 K, t = 3 h.

RCl	[Cl^−^] [mmol g^−1^]	S_BET_ [m^2^ g^−1^]	Color
CH_3_Cl	0.17	104	Colorless
CH_2_Cl_2_	0.21	90	Colorless
CHCl_3_	0.27	94	Colorless
CCl_4_	0.15	89	Colorless
EtCl	0.17	32	Colorless
*n*-BuCl	0.24	95	Dark yellow
*i*-BuCl	0.18	--	Colorless
*s*-BuCl	0.84	69	Colorless
*t*-BuCl	19.31	9	Pink
*t*-AmCl	12.22	--	Pink

**Table 7:** Properties of MgO after its reaction with various alkyl bromides. T = 373 K, t = 3 h.

RBr	[Br^−^] [mmol g^−1^]	S_BET_ [m^2^ g^−1^]	Color
CH_3_Br	0.21	97	Colorless
CH_2_Br_2_	0.17	91	Pale yellow
CHBr_3_	0.23 ^1^	105	Beige
EtBr	0.18	58	Pale yellow
*n*-BuBr	0.28	72	Dark yellow
*i*-BuBr	0.22	--	Pale yellow
*s*-BuBr	3.49	4	Pale pink
*t*-BuBr	12.42	1	Violet

^1^ 0.24 mmol g^−1^ of Br^−^ after 6 h.

**Table 8:** Properties of MgO after its reaction with various alkyl iodides. T = 373 K, t = 3 h.

RI	[I^−^] [mmol g^−1^]	S_BET_ [m^2^ g^−1^]	Color
CH_3_I	0.25 ^1,2,3,4^	85	Pale yellow
CH_2_I_2_	0.08 ^5^	--	Yellow
CHI_3_	0.10 ^6^	--	Yellow
EtI	0.23 ^7^	89	Pale yellow
*n*-BuI	0.28	88	Pale yellow
*i*-BuI	0.27	91	Yellow
*s*-BuI	0.56	41	Yellow
*t*-BuI	2.33 ^8^	15	Yellow-red

^1^ 0.06 mmol g^−1^ of I^−^ at 293 K for 1 h; ^2^ 0.16 mmol g^−1^ of I^−^ at 373 K for 1 h; ^3^ 0.27 mmol g^−1^ of I^−^ and also 0.006 mmol g^−1^ of I_2_ at 373 K for 6 h; ^4^ 0.24 mmol g^−1^ of I^−^ and also 0.015 mmol g^−1^ of I_2_ at 473 K for 3 h; ^5^ 0.07 mmol g^−1^ of I^−^ at 373 K for 1 h; ^6^ 0.05 mmol g^−1^ of I_2_ for 10 h; ^7^ 0.25 mmol g^−1^ of I^−^ for 6 h; ^8^ 1.58 mmol g^−1^ of I^−^ for 20 min.

**Table 9:** Concentrations of X^−^ ions on the surface of MgO after its reaction with various alkyl dihalides or interhalogen compounds. T = 373 K, t = 3 h.

RX_2_ or IX_n_	[X^−^] [mmol g^−1^]			Color
	[Cl^−^]	[Br^−^]	[I^−^]	
ClCH_2_Br	0.06	0.19	--	Colorless
ClCH_2_I	0.20	--	0.04	Yellowish
BrCH_2_I	--	0.17	- ^1^	Yellow
Cl(CH_2_)_3_Br	0.10	0.18	--	Colorless
Br_2_	--	0.12 ^2^	--	Yellowish
I_2_	--	--	0.01 ^3^	Yellow
IBr	--	0.19	0	Yellow–brown
ICl_3_	0.10	--	0	Colorless

^1^ less than 0.005 mmol g^−1^; ^2^ 0.005 mmol g^−1^ of Br_2_. Data taken from Table 1. ^3^ and 0.19 mmol g^−1^ of I_2_ [21].

**Table 10:** Strength of acidic and basic sites on the surface of MgO-X_2_ (X = Br or I) and MgO-HX (X = Cl, Br or I). [X^−^] = 200 μmol g^−1^ of MgO.

Modifier	S_BET_ [m^2^∙g^−1^]	Strength of Sites	
		Acidic	Basic
*--*	100	H_0_ > 4.8	7.2 ≤ H_-_ < 33.0
Br_2_	98	0.8 < H_0_ ≤ 4.8	7.2 ≤ H_-_ < 15.0
I_2_	100	−3.0 < H_0_ ≤ 4.8	7.2 ≤ H_-_ < 9.3
HCl	95	−5.6 < H_0_ ≤ 4.8	7.2 ≤ H_-_ < 18.4
HBr	96	−3.0 < H_0_ ≤ 4.8	7.2 ≤ H_-_ < 18.4
HI	98	−3.0 < H_0_ ≤ 4.8	7.2 ≤ H_-_ < 18.4

**Table 11:** Strength of acidic and basic sites on the surface of magnesium oxide modified with mono- and polychloroderivatives of methane.

Modifier	S_BET_ [m^2^∙g^−1^]	Strength of Sites	
		Acidic	Basic
CH_3_Cl	104	0.8 < H_0_ ≤ 4.8	7.2≤ H_-_ < 22.3
CH_2_Cl_2_	90	0.8 < H_0_ ≤ 4.8	7.2 ≤ H_-_ < 22.3
CHCl_3_	94	−3.0 < H_0_ ≤ 4.8	7.2 ≤ H_-_ < 22.3
CCl_4_	98	H_0_ > 4.8	7.2 ≤ H_-_ < 33.0

**Table 12:** Strength of acidic and basic sites on the surface of MgO-RX catalysts.

RX	S_BET_ [m^2^∙g^−1^]	Strength of Sites	
		Acidic	Basic
MeCl	104	0.8 < H_0_ ≤ 4.8	7.2 ≤ H_-_ < 22.3
*t*-BuCl	9	0.8 < H_0_ ≤ 4.8	H_-_ < 7.2
MeBr	97	0.8 < H_0_ ≤ 4.8	7.2 ≤ H_-_ < 15.0
EtBr	58	0.8 < H_0_ ≤ 4.8	7.2 ≤ H_-_ < 18.4
MeI	91	−3.0 < H_0_ ≤ 4.8	7.2 ≤ H_-_ < 9.3
EtI	19	0.8 < H_0_ ≤ 4.8	7.2 ≤ H_-_ < 15.0

**Table 13:** Catalytic activity of MgO-I_2_ (MeOH) catalysts, [I_2_] = 0–200 μmol∙g^−1^, in transfer hydrogenation reaction between ethanol and acrolein. Donor–acceptor molar ratio D/A = 6.

[I_2_] [μmol∙g^−1^]	T [K]	Conversion [%]	Moles from 100 Moles of Acrolein			
			UOL	SAL	SOL	Others
0	473	15	15	0	0	0
	523	32	31	1	0	0
	573	51	47	2	1	1
50	473	45	45	0	0	0
	523	68	65	0	2	1
	573	69	69	1	5	1
100	473	67	65	0	1	1
	523	78	73	0	4	1
	573	82	67	1	9	1
200	473	73	57	0	3	13
	523	78	48	1	6	23
	573	82	47	1	10	24
100 ^1^	473	67	64	0	2	1
	523	78	67	1	5	5
	573	83	66	1	9	7

^1^ EtOH as a reagent and solvent.

**Table 14:** Catalytic activity of MgO-Br_2_ (MeOH) catalysts, [Br_2_] = 0–300 μmol∙g^−1^, in transfer hydrogenation reaction between ethanol and acrolein. Donor–acceptor molar ratio D/A = 6.

[Br_2_] [μmol∙g^−1^]	T [K]	Conversion [%]	Moles from 100 Moles of Acrolein			
			UOL	SAL	SOL	Others
0	473	15	15	0	0	0
	523	32	31	1	0	0
	573	51	47	2	1	1
100	473	63	63	0	0	0
	523	77	75	0	1	1
	573	82	74	1	5	2
200	473	72	72	0	0	0
	523	81	76	0	2	3
	573	91	46	0	13	32
300	473	63	58	0	1	4
	523	82	41	0	9	32
	573	86	37	0	11	38

**Table 15:** Catalytic activity of MgO-HX (X = Cl, Br or I) [HX] = 200 μmol∙g^−1^ in transfer hydrogenation reaction between ethanol and acrolein. Donor–acceptor molar ratio D/A = 6.

HX	T [K]	Conversion [%]	Moles from 100 Moles of Acrolein			
			UOL	SAL	SOL	Others
HCl	473	35	34	0	0	1
	523	56	50	0	1	5
	573	80	58	1	6	15
HBr	473	64	62	0	1	1
	523	69	60	1	1	7
	573	75	54	0	3	18
HI	473	62	60	0	1	1
	523	73	70	0	2	1
	573	74	63	1	5	5
^1^	473	18	18	9	0	0
	523	34	32	2	0	0
	573	53	46	4	2	1

^1^ MgO-H_3_PO_4_, [H_3_PO_4_] = 200 μmol g^−1^, for comparison.

**Table 16:** Catalytic activity of MgO-RX in transfer hydrogenation reaction between ethanol and acrolein. Donor–acceptor molar ratio D/A = 6.

RX	T [K]	Conversion [%]	Moles from 100 Moles of Acrolein			
			UOL	SAL	SOL	Others
--	473	15	15	0	0	0
	523	32	31	1	0	0
	573	51	47	2	1	1
MeI	473	69	65	1	2	1
	523	76	58	2	15	1
	573	81	53	4	21	3
EtCl	473	45	44	0	0	1
	523	65	57	0	2	6
	573	86	43	0	4	38
EtBr	473	75	73	0	1	1
	523	83	77	0	3	3
	573	87	62	0	11	14
EtI	473	73	70	0	2	1
	523	77	65	1	10	1
	573	80	60	2	15	3

## Data Availability

All of the data are available in the manuscript.

## Supplementary Materials

The following supporting information can be downloaded at: https://www.mdpi.com/article/10.3390/molecules29133180/s1. Table S1. Crystallite size (D) of the MgO phase in MgO-RX catalysts. D = 11.9 nm for MgO; Table S2. Crystallite size (D) of phases other than the MgO phase in MgO-RX catalysts.
