# Supplementary material for: MgO Modified by X2, HX, or Alkyl Halide (X = Cl, Br, or I) Catalytic Systems and Their Activity in Chemoselective Transfer Hydrogenation of Acrolein into Allyl Alcohol
Source: Molecules. 2024 Jul 3;29(13):3180. doi: 10.3390/molecules29133180 (PMC11243119; doi:10.3390/molecules29133180)
Supplement: Supplementary file 1 [file molecules-29-03180-s001.zip › molecules-3055832-supplementary.pdf]

Supplementary Materials

Table S1. Crystallite size (D) of the MgO phase in MgO-RX catalysts. D = 11.9 nm for MgO.

RCl	D [nm]	RBr	D [nm]	RI	D [nm]
MeCl	12.3	MeBr	12.0	MeI	12.0
n-BuCl	13.7	*n*-BuBr	12.7	*n*-BuI	13.0
s-BuCl	14.2	*s*-BuBr	13.2	*s*-BuI	15.3
t-BuCl	10.0	*t*-BuBr	--	*t*-BuI	11.8

Table S2. Crystallite size (D) of phases other than the MgO phase in MgO-RX catalysts.

RX	Phase	D [nm]
s-BuBr	$MgBr_2{\cdot}6H_2O$	27.0
t-BuBr	$MgBr_2{\cdot}6H_2O$	25.5
t-BuCl	$MgCl_2{\cdot}6H_2O$	22.0
